# Interest of a Commercialized *Pneumocystis jirovecii* Quantitative PCR to Discriminate Colonization from *Pneumocystis* Pneumonia according to the Revised EORTC/MSGERC Criteria

**DOI:** 10.3390/jcm12010316

**Published:** 2022-12-31

**Authors:** Florian Lussac-Sorton, Tara Fleur, Thibault Voisin, Nahéma Issa, Élodie Blanchard, Éléna Charpentier, Laurence Delhaes

**Affiliations:** 1 Service de Parasitologie-Mycologie, Groupe Hospitalier Pellegrin, CHU de Bordeaux, 33000 Bordeaux, France; 2 Eurofins, Polyclinique Bordeaux Nord, 18 Rue Henri Guillemin, 33300 Bordeaux, France; 3 Réanimation Médicale, Groupe Hospitalier Saint-André, CHU de Bordeaux, 33000 Bordeaux, France; 4 Service de Pneumologie, Hôpital Haut-Lévêque, Groupe Hospitalier Sud, CHU de Bordeaux, 33600 Pessac, France

**Keywords:** *Pneumocystis jirovecii*, PCP, colonization, qPCR

## Abstract

Quantitative PCR (qPCR) is highly sensitive to diagnose *Pneumocystis jirovecii* (*Pj*) pneumonia (PCP). However, differentiating PCP and colonization remains difficult. This study aimed to establish the performances of the commercialized qPCR MycoGENIE^®^ *Pj* kit (Ademtech) to distinguish PCP and *Pj* colonization. Patients with a positive *Pj* qPCR on bronchoalveolar lavage (BAL) or upper respiratory tract (URT) samples were prospectively included between May 2019 and December 2020 at Bordeaux University Hospital. They were classified in “PCP” or “*Pj* colonization” groups based on the revised EORTC/MSGERC criteria. The two groups’ results were compared; ROC curves were produced to determine the best thresholds. Excluding the low number of HIV-positive subjects, there were 100 PCP (32 BAL, 68 URT) and 70 *Pj* colonization (34 BAL, 36 URT). *Pj* loads were significantly higher in PCP compared to *Pj* colonization group (*p* ≤ 0.01). The best cut-offs for PCP diagnosis were 31.45 Cq/8275 copies/mL for BAL and 32.33 Cq/8130 copies/mL for URT (sensitivity = 59.4%, 63.3%, specificity = 82.4%, 88.9%, respectively). Fungal load quantification using MycoGENIE^®^ *Pj* qPCR helps discriminating PCP from colonization, high fungal loads being indicative of probable PCP. Low load results should be interpreted with caution, in accordance with clinical and radiological signs.

## 1. Introduction

*Pneumocystis jirovecii* (*Pj*) is an opportunistic fungus responsible for severe pneumonia in patients with various immunosuppressive conditions affecting lymphocytes-mediated immunity [1,2,3]. The main risk factor leading to *Pneumocystis* pneumonia (PCP) has long been advanced HIV infection with a CD4 T lymphocyte count lower than 200 cells/mm^3^, but its incidence has decreased since the introduction of chemoprophylaxis and highly active antiretroviral therapy. PCP now mainly happens in non-HIV immunocompromised patients, such as patients with solid or hematopoietic malignancies and patients with long-lasting immunosuppressive treatments for a solid organ transplant or a hyper-inflammatory disease [1,2,4]. These patients exhibit a more acute and severe clinical presentation and lower fungal loads in their respiratory tracts compared to those infected with HIV [5]. However, low fungal loads cause diagnostic difficulties considering the inability to culture *Pj* in vitro and the lack of sensitivity of microscopic examination of respiratory samples in this context [6]. *Pj* quantitative PCR (qPCR) has grown to be an indispensable tool for PCP diagnosis given its high sensitivity [7]. Hence, a negative PCR result on bronchoalveolar lavage (BAL) allows for ruling out PCP with a predictive negative value close to 100% [8]. It allows the detection of minor traces of *Pj* DNA (hundreds of genome copies/mL) in respiratory samples, revealing PCP disease as well as *Pj* colonization [9].

Indeed, asymptomatic colonization is being increasingly identified using *Pj* qPCR, in numerous populations considered as immunocompetent throughout the world (ranging from 15% to 70%) and with a particularly high prevalence among children [10,11,12]. This highlights the role of *Pj* colonization as a major reservoir of infections (especially of nosocomial PCP), and as a comorbidity factor of certain diseases, especially chronic obstructive pulmonary disease (COPD) [11,13,14].

However, *Pj* pulmonary colonization is also encountered in immunosuppressed subjects, sometimes complicating PCP diagnosis in case of positive qPCR. Therefore, fungal load quantification or semi-quantification has been proposed to help physicians in discriminating active PCP from *Pj* colonization with mitigate results [15]. However, the lack of standardization between qPCR assays (whether in-house or commercialized assays) in terms of sample preparation, DNA extraction, targeted gene, and PCR method prevents the application of consensual cut-off values.

In this context, this study aimed to evaluate the commercialized MycoGENIE^®^ *Pneumocystis jirovecii* Real-Time PCR kit (Ademtech, Pessac, France) which includes a quantification range, in its ability to distinguish PCP from colonization. The *Pj* qPCR quantification results have thus been compared between the two groups (PCP or colonization patient groups) established on the basis of the 2021 revised EORTC/MSGERC criteria for PCP [4]. Mainly, this study aimed to determine a fungal load threshold between PCP and *Pj* colonization using this qPCR kit, and to evaluate the test performances with these cut-off values.

## 2. Materials and Methods

### 2.1. Patients and Samples 

All prospectively included patients had a positive MycoGENIE^®^ *Pneumocystis jirovecii* qPCR, performed on a respiratory sample between May 2019 and December 2020 at Bordeaux University Hospital. Exclusion criteria were patients under 18 years old and a preventive or curative treatment for PCP more than one day before the date of the concerned respiratory sampling. Due to the well-known significant difference between the fungal load of HIV-positive and HIV-negative patients, we decided to split them for two distinct analyses [5]. A distribution depending on the sample’s nature was also performed; BAL and upper respiratory tract (URT) samples were analyzed separately considering the recognized higher sensitivity of BAL (current gold standard sample) [7,15]. URT samples included sputa and tracheobronchial aspirates as previously reported [8,16]. 

### 2.2. Ethics 

All included patients were informed about the anonymous use of their medical chart data for clinical research purposes. The samples were collected from research subjects, during the course of routine care management at the discretion of the clinician, and patient information was anonymized prior to analysis. Therefore, this protocol did not require approval from an ethics committee and was exempt from formal informed consent. It complies with the ethical and legal requirements of French law (15 April 2019) and the Declaration of Helsinki.

### 2.3. Patients’ Classification

**Figure 1:**
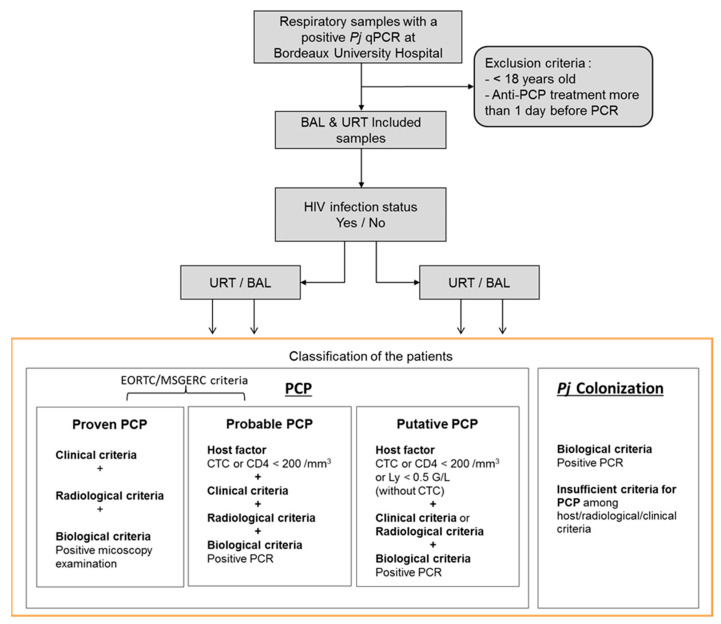
Flow chart of patients’ inclusion and classification. *Pj*: *Pneumocystis jirovecii*; PCP: *Pneumocystis* pneumonia; BAL: bronchoalveolar lavages; URT: upper respiratory tract; CTC: corticosteroids; CD4: CD4 T lymphocytes; Ly: total lymphocytes.

The patients were classified in PCP or *Pj* colonization group according to the revised EORTC/MSGERC criteria [4], as follows: a classification in the PCP group requires the triad of host factors, clinical/radiological arguments, and mycological arguments with a categorization into two subgroups, “proven PCP” and “probable PCP,” based on a positive or negative microscopic examination result with conventional staining (Figure 1).

In addition to these two subgroups defined by EORTC/MSGERC criteria, a “putative PCP” group was added in this study, in order to take into account the immunosuppressed patients without corticosteroids (CTC, at doses in agreement with EORTC/MSGERC criteria [4]) who did not have a CD4 T cells blood count and patients who did not have a radiological examination (as requested by the EORTC/MSGERC criteria [4]). In this “putative PCP” group, the host factor could be a lymphocyte total count <0.5 G/L, and there could be only one of the clinical and radiological arguments in addition to the mycological argument. Hence, in the present study, the PCP group includes all three “proven PCP,” “probable PCP,” and “putative PCP” categories. All remaining patients were classified in the “*Pj* colonization” group. Importantly and in concordance with EORTC/MSGERC criteria, the fungal load was never taken into account for this classification.

### 2.4. Clinical and Radiological Data

We collected retrospectively the clinical histories of all the patients. The collected data included the following: age, sex, underlying diseases (solid organ transplantation, inflammatory disease, hematologic malignancy, solid malignancy, allogeneic hematopoietic stem cell transplantation (HSCT), HIV status), pertinent clinical symptoms at diagnosis (fever, dyspnea, dry cough, hypoxemia (PaO_2_ < 80 mmHg)), presence of typical radiological features on chest radiograph or computed tomography scan (diffuse interstitial infiltrates with ground-glass opacities), use of anti-*Pneumocystis* treatment after sampling, immunosuppressive treatment (especially the use of CTC), and clinical evolution and outcome (death or not) in the 6 weeks after diagnosis. Biological data including total lymphocyte and CD4 T cells blood counts were collected within a maximum of 7 days before and after the respiratory sample for *Pj* PCR was performed.

### 2.5. Pre-Treatment and Microscopic Examination of Respiratory Samples

Unlike BAL, URT samples were first homogenized for 30 min at 37 °C with Dithiothreitol (Sputasol^®^, Oxoid, France) in a 1:1 (*v*:*v*) ratio. After a centrifugation step (1200 g/min, 10 min), each sample was submitted to microscopic examination to detect fungal cysts and/or trophozoites using Musto and May-Grünwald-Giemsa staining [3].

### 2.6. DNA Extraction and qPCR Assay

A volume of 250 µL pre-treated pellet was added to 250 µL of Cobas^®^ lysis buffer (Roche, Bâle, Switzerland) before performing DNA extraction. DNA extraction and amplification were carried out using the automated ELITe InGenius^®^ system (ELITechGroup, Puteaux, France). DNA extraction was performed using ELITe InGenius^®^ SP 200 kit (ELITechGroup, Puteaux, France) and *Pj* DNA amplification using MycoGENIE^®^ *Pneumocystis jirovecii* (Ademtech, Pessac, France), according to the manufacturer instructions. MycoGENIE^®^ *Pneumocystis jirovecii* assay is a real-time PCR kit targeting the *P. jirovecii* mitochondrial large sub-unit rRNA gene (mtLSU) that includes a quantification standard range to estimate the fungal load of each sample in genome copy number/mL and an internal inhibition control. Of note, this kit is CE-IVD marked. Results of fungal loads were expressed as quantification cycles (Cq, a positive result being defined as Cq < 38 according to the manufacturer instructions) and genome copies/mL.

### 2.7. Statistics

Descriptive analyses have been performed to summarize the studied patient population. Continuous variables were presented as medians with interquartile range (25th–75th percentile, IQR). Qualitative variables were expressed as absolute numbers and percentages (%). The Mann–Whitney, Kruskal–Wallis (and subsequent Dunn’s test in case of significant difference), and the χ^2^ tests were used when appropriate. Alternatively, the Fisher’s test was used for a low number of patients. Receiver operator characteristic (ROC) curves were used to define the best cut-off values for BAL and URT samples. All the statistical analyses were performed using the software GraphPad Prism (version 5.1 for Windows; GraphPad Software, San Diego, CA, USA). *p* < 0.05 was considered statistically significant.

## 3. Results

Between May 2019 and December 2020, there were 253 patients with a positive MycoGENIE^®^ *Pneumocystis jirovecii* qPCR on a respiratory sample. Among them, 66 were excluded because they were under 18 years old (*n* = 23) or had received an anti-*Pj* treatment before sampling (*n* = 43). On the 187 remaining patients, there were 170 HIV-negative patients and 17 HIV-positive patients. Given the limited number of HIV-positive patients, the added value of this *Pj* quantification for PCP diagnosis was evaluated on the HIV-negative patient population. Among them, 66 had positive BAL samples and 104 had positive URT samples (88 sputa, 16 tracheobronchial aspirates). Concerning the BAL, thirty-two (48%) were from patients classified in the PCP group (four as “proven PCP”, seventeen as “probable PCP,” and eleven as “putative PCP”) while among URT samples, sixty-eight (65%) were from patients classified in the PCP group (four as “proven PCP”, forty-six as “probable PCP,” and eighteen as “putative PCP”) (Figure 1).

Epidemiological, clinical, radiological, and biological data are summarized in Table 1. Regarding immunosuppression conditions, patients with solid malignancies were more present in the PCP group than in the *Pj* colonization group for URT. Regarding clinical symptoms, hypoxemia was significantly more frequent in the PCP group for both BAL and URT (*p* = 0.007 and *p* = 0.004, respectively), as was fever (*p* = 0.02 for BAL, *p* = 0.02 for URT) and dyspnea for URT (*p* < 0.0001). As expected, the group of patients with PCP received significantly more curative anti-*Pj* treatment after the positive qPCR result compared to the colonized patients (*p* < 0.0001 for both sample types), reflecting the physicians’ decision. Of note, about 25% of patients classified in the *Pj* colonization group received an anti-*Pj* curative treatment. The characteristics of the included patients according to their subgroup of PCP (proven, probable, or putative PCP) are detailed in Appendix A (Table A1).

**Figure 2:**
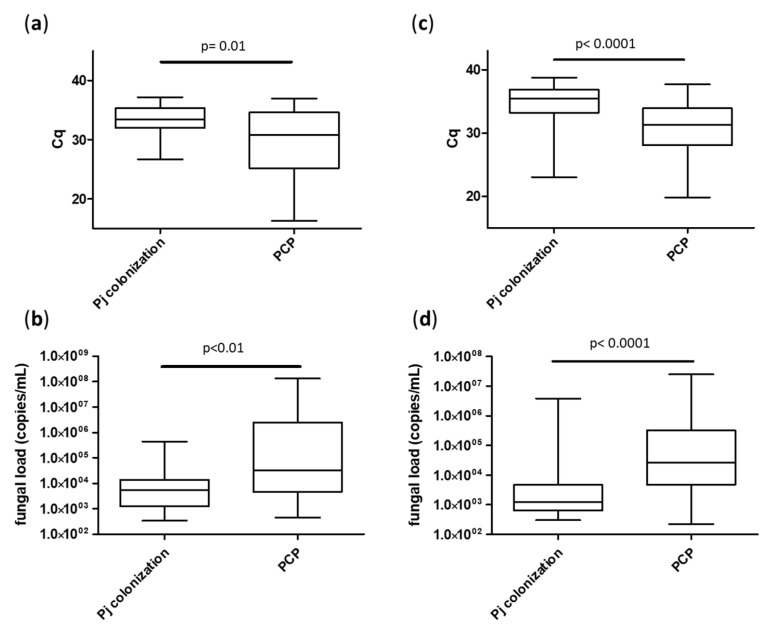
Median fungal loads according to patient groups (*Pj* colonization or PCP), sample types: BAL (**a**,**b**) and URT (**c**,**d**) and expressed in Cq (**a**,**c**) or in *Pj* genome copies/mL (**b**,**d**). Cq: quantification cycle.

**Figure 3:**
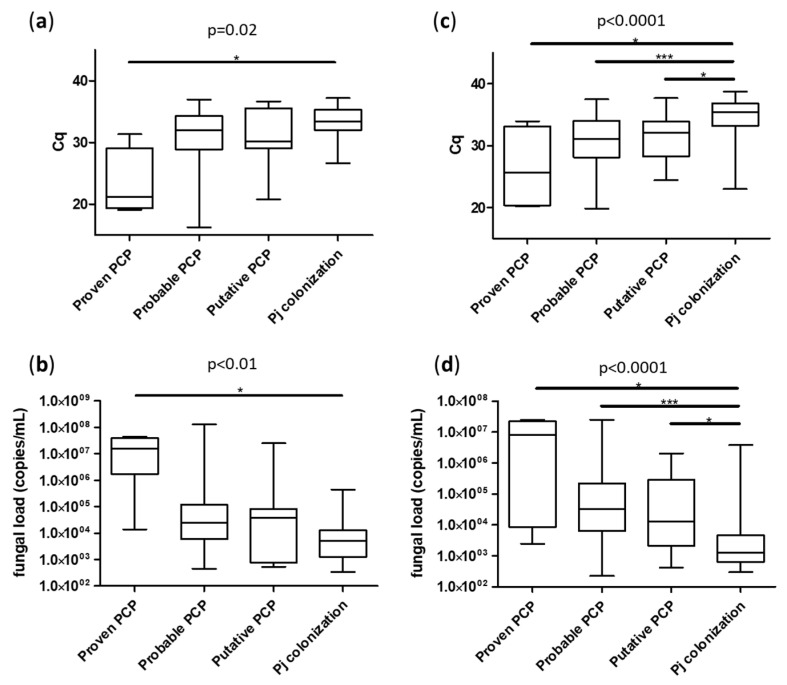
Median fungal loads according to patient classification with EORTC/MSGERC criteria, sample types: BAL (**a**,**b**) and URT (**c**,**d**) and expressed in Cq (**a**,**c**) or in *Pj* genome copies/mL (**b**,**d**). Cq: quantification cycle. *: *p* < 0.05 in Dunn’s test; ***: *p* < 0.001 in Dunn’s test. Only significant differences are represented by lines.

Focusing on molecular diagnosis, fungal loads were significantly higher in the PCP group (median Cq = 30.8 [25.2; 34.7] for BAL and 31.2 [28.1; 33.9] for URT) compared to the *Pj* colonized group (median Cq = 33.4 [32.0; 35.3] for BAL and 35.4 [33.2; 36.9] for URT), *p* < 0.01 (Figure 2). As expected, *Pj* loads were significantly higher in the sub-group of “proven PCP” compared to either “probable PCP” or “putative PCP” for both sample types (Figure 3).

**Figure 4:**
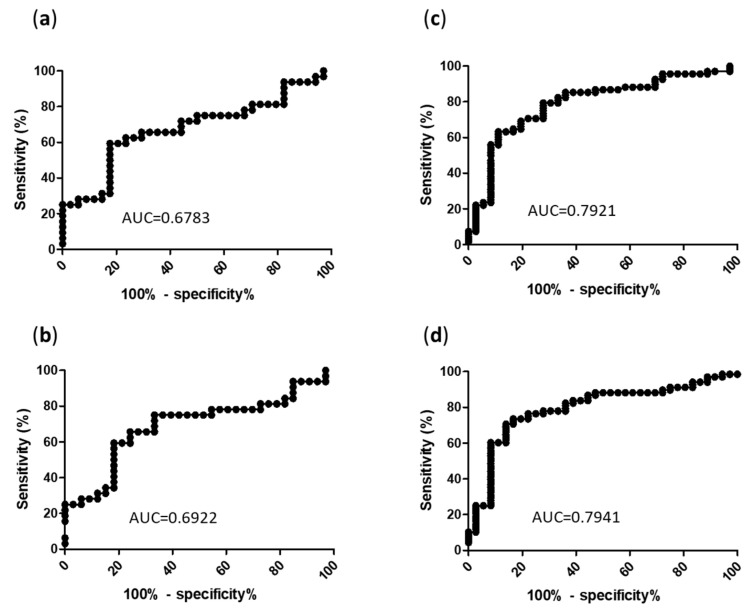
ROC curves of MycoGENIE^®^ qPCR for the diagnosis of PCP according to sample type. (**a**) Cq in BAL; (**b**) *Pj* genome copies/mL in BAL; (**c**) Cq in URT; (**d**) *Pj* genome copies/mL in URT; AUC: area under the curve; Cq: quantification cycle.

To determine the best cut-off values to discriminate PCP from *Pj* colonization, ROC curves were produced to display the fungal load ability of binary (PCP versus *Pj* colonization) classifiers for each sample type (BAL and URT). The area under the ROC curves for Cq were 0.678 for BAL and 0.792 for URT (Figure 4). The qPCR cut-off values (expressed as Cq and fungal loads) providing the best sensitivities and specificities are summarized in Table 2. Cq values corresponding to a 100% sensitivity were 37.08 for BAL (specificity = 2.9%) and 38.21 for URT (specificity = 2.6%). Cq values corresponding to a 100% specificity were 25.4 for BAL (sensitivity = 25.0%) and 22.55 for URT (sensitivity = 7.35%). Consequently, the optimal Cq values allowing for the discrimination of PCP from *Pj* colonization were 31.45 for BAL (sensitivity = 59.4%, specificity = 82.4%) and 32.33 for URT (sensitivity = 63.2%, specificity = 88.9%). The putative PCP group having been added to the original EORTC/MSGERC classification by us (see Section 2.3), the thresholds for URT and BAL have also been evaluated without this group (PCP = proven PCP + probable PCP) leading to the same optimal Cq values: 31.45 for BAL (sensitivity = 60.9%, specificity = 82.4%) and 32.33 for URT (sensitivity = 64.7%, specificity = 88.9%).

As expected, BAL quantification exhibited higher fungal loads compared to URT samples, in agreement with *Pj* alveolar tropism. However, URT ROC curve showed accurate estimate (Table 2).

## 4. Discussion

In this study, we aimed to evaluate the added value of *Pj* pulmonary quantification using the MycoGENIE^®^ qPCR for discriminating PCP and *Pj* colonization. The fungal load was significantly higher in the group of patients diagnosed with PCP compared to the group of colonized subjects, for both BAL and URT samples. However, the determination of threshold values for a clinical interpretation was not fully suitable. Thresholds with a 100% specificity were obtained for extreme fungal loads (millions of genome copies/mL) on both sample types, yet they were associated with highly poor sensitivities (27.3% for BAL and 8.8% for URT). On the opposite, a perfect sensitivity was associated with thresholds as low as only hundreds of genome copies/mL that were not helpful for PCP diagnosis (2% specificity) (Table 2). The best thresholds as predicted by ROC curves analysis for BAL (31.45 Cq or 8275 genome copies/mL) and for URT (32.33 Cq or 8130 genome copies/mL) had notable specificities around 85% (82.4% and 88.9% respectively) with moderate sensitivities around 60% (59.4% and 63.2%, respectively).

From these results, quantifying pulmonary *Pj* load remains of interest to determine PCP mostly when fungal load is high (as in case of millions of genome copies). However, in case of moderate or low loads, excluding a PCP is not possible and requires a global evaluation with the clinical/radiological context. A system of two thresholds has been proposed in previous works on *Pj* qPCR: one low threshold under which *Pj* colonization would be highly probable and a high threshold over which a PCP is greatly probable, and a gray-zone in-between that requires an interpretation with the global clinical and radiological context of the patient [6,16,17], but it was not adequate in our study given the thresholds values obtained (Table 2). This difficulty in interpreting low *Pj* loads is in concordance with previous reports of qPCR evaluations [9,16,18] and could be explained by different factors.

Firstly, a disconnection between the fungal load and PCP severity has been reported in humans and in rodent models. Indeed, the pulmonary symptoms would be related to an overstated and inadequate immune host response rather than to the fungus itself [5,19,20,21]. Thus, low fungal loads could be associated with a real PCP diagnosis. On the other hand, very high fungal loads may reflect the incapacity of the host to control the fungus expansion, hence an unsuitability with a colonization state. 

Secondly, an inter-strain variability of the copy number of the genes targeted by the qPCR (including mtLSU targeted by MycoGENIE^®^ *Pneumocystis jirovecii*) has been described [9]. This limits the correlation between the fungal load estimated from the qPCR and the actual *Pj* load in the lungs, and can consequently reduce the efficiency of such quantifications to predict PCP diagnosis [22].

Finally, the colonization and the disease state (PCP) are currently presented as a continuum rather than two distinct states; *Pj* colonization being largely considered as a “pre-PCP” state in immunocompromised patients. Furthermore, as observed in our study, patients considered as colonized by physicians often received a *Pj* prophylactic treatment to avoid a potential evolution toward PCP. Hence, the detection of these colonized patients can also be an advantage in preventing possible further complications such as a PCP occurrence and a nosocomial *Pj* dissemination [15].

Of note, microscopic examination after staining was only positive in 6% of PCP cases, confirming the major role of qPCR in PCP diagnosis despite some interpretation difficulties related to the *Pj* colonization status. Use of immunofluorescent assay could have improved the performances of the microscopic examination given its superior sensitivity to conventional staining [4,8]; however, this technique is not routinely used in Bordeaux University Hospital.

This study has some limitations. Firstly, we selected patients and samples according to a stringent cut-off based on prolonged PCP treatment/prophylaxis (more than one day before the sampling date) to assess efficiently the PCR performances. Given the low number of HIV-positive patients excluding a significant statistical analysis, this population was not analyzed. Although BAL is consensually considered as the reference sample for PCP diagnosis [7], the number of BAL samples in our study was limited compared to URT samples, which has mitigated the power of the statistical analysis and the threshold determination. This was related to the clinical care habits in Bordeaux University Hospital, in relation to the risks associated with BAL sampling, especially in patients with unstable respiratory states. However, both type of respiratory samples exhibited fungal loads significantly different between PCP and *Pj* colonization groups, and both leading to interpretation cut-off values. Finally, β-D glucan blood concentration was not available in this study. While this indirect biomarker can help differentiating *Pj* colonization and PCP, and has been associated with results of qPCR quantification to diagnose PCP [23], it is not routinely used at Bordeaux University Hospital. As recently recommended, we classified the patients according to the newly revised EORTC/MSGERC adapted for PCP [4]. The use of this EORTC/MSGERC criteria allowed us a more neutral classification in comparison to the decision of the patients’ physicians. In this latter case, the physicians could over-estimate the PCP diagnosis and over-treat in fear of missing a disease of which mortality is about 20 to 30%. Also, physicians are often informed of the fungal load prior to establishing a PCP diagnosis and may consider it for patient classification. This EORTC/MSGERC classification represents also a more standardized method enabling the comparison of results with other studies, as opposed to groups of experts each having their own definitions of PCP [6,16,17]. However, we added in our study a third subgroup of PCP named “putative PCP” to the two existing categories “proven PCP” and “probable PCP” defined by the EORTC/MSGERC classification, in order to include the immunosuppressed patients without CTC who did not have any measure of blood CD4 T cells counts or the subjects who did not have a radiological examination. Indeed, the EORTC/MSGERC classification is based on the presence of four criteria: host immunosuppression, clinical features, radiological features, and mycological arguments. The host factor includes all patients receiving high doses of CTC, which constitutes nowadays a large majority of patients with PCP, while a CD4 T cells blood count below 200 cells/mm^3^ is required for all other types of immunosuppression [4]. However, HIV-negative patients from oncology or inflammatory diseases do not always receive CTC and/or have a CD4 T cells blood count in their routine care (i.e., 50% to 80% of missing values in our cohort, Table 1). Moreover, in HIV-negative patients, the CD4 T cells threshold is not as predictive as in HIV-positive patients, and PCP can develop while the CD4 T cells are over 200 cells/mm^3^ [24], leading us to propose a third “putative PCP” subgroup. In addition, we also included tracheobronchial aspirates among URT samples, because it is frequently performed in Bordeaux University Hospital clinical care habits [25] and was previously reported for PCP molecular diagnosis [8,16,25], even if this sample type is not mentioned in the EORTC/MSGERC criteria.

Children (23 in our cohort) were excluded from these analyses to avoid the cases of primo-infections that could be associated with different fungal loads in comparison to the mechanism of reinfections that is now largely accepted for PCP pathophysiology in adults [26]. Patients receiving prophylactic or curative anti-*Pj* treatment before the qPCR were also excluded from the study since their fungal load could have been modified under treatment.

Finally, our population of HIV-negative patients exhibited characteristics similar to populations previously reported: median age about 65 years old; various immunosuppressive conditions including mainly transplanted patients; a large population of solid and hematological cancers and inflammatory diseases; about 70% of patients receiving corticosteroids [5,27,28]. 

To conclude, quantification of fungal loads using MycoGENIE^®^ *Pneumocystis jirovecii* qPCR helps to discriminate PCP from *Pj* colonization in our experience, with Cq < 31.45 (or loads > 8275 genome copies/mL) for BAL and Cq < 32.33 (or loads > 8130 genome copies/mL) for URT samples being indicative of PCP. However, as observed in previous works on qPCR, low fungal load results, being possibly associated with either PCP or colonization, should be interpreted with caution, in accordance with clinical and radiological signs.

## Figures and Tables

**Table 1:** Epidemiological, clinical, radiological, and biological features of the HIV-negative population.

Features	Distribution of Patients According to Respiratory Sample Type and *Pj* Status (PCP or Colonization)					
	BAL (*n* = 66)			URT (*n* = 104)		
	Colonization (*n* = 34)	PCP (*n* = 32)	*p*	Colonization (*n* = 36)	PCP (*n* = 68)	*p*
**Median age** **in years** **[min–max]**	61 [21–87]	68 [37–83]	0.01	72 [42–88]	70 [30–94]	0.86
**Sex ratio** **M/W (n/n)**	2.09 (23/11)	0.88 (15/17)	0.09	1.60 (22/14)	1.29 (37/31)	0.51
**Immunosuppressive condition** **(n (%))**	20 (58.8)	32 (100)	0.0004	22 (61)	68 (100)	0.015
**SOT**	3 (8.8)	6 (18.8)	0.30	6 (16.7)	23 (33.8)	0.06
**Hematologic malignancy**	3 (8.8)	7 * (21.8)	0.20	7 (19.4)	9 ** (13.2)	0.40
**Solid malignancy**	10 (29.4)	15 (46.9)	0.14	2 (5.6)	18 (26.5)	0.01
**Inflammatory disease**	4 (11.8)	3 (9.4)	1.0	5 (13.9)	11 (16.2)	0.76
**Other**	0 (0)	1 (3.1)	0.48	2 (5.6)	7 (10.3)	0.49
**Total lymphocytes count** **G/L [IQR]**	1.26 [0.92–1.64]	0.53 [0.30–0.76]	<0.001	0.96 [0.61–1.43]	0.71 [0.39–1.11]	0.09
**CD4 T lymphocytes count < 200 cells/mm^3^** **(n/total (%))**	1/9 (11.1)	7/11 (63.6)	0.03	0/6 (0)	12/25 (48)	0.06
**Use of CTC (n (%))**	5 (14.7)	23 (71.9)	<0.0001	13 (36.1)	45 (66.2)	0.003
**Clinical/radiological features** **(n, (%))**						
**Hypoxemia**	9 (26.5)	19 (59.4)	0.007	10 (27.8)	39 (57.4)	0.004
**Dyspnea**	18 (52.9)	23 (71.9)	0.11	19 (52.8)	57 (83.8)	<0.0001
**Fever (>38.3 °C)**	13 (38.2)	21 (65.6)	0.01	16 (44.4)	46 (67.6)	0.02
**Dry cough**	5 (14.7)	8 (25)	0.29	8 (22.2)	20 (29.4)	0.43
**Typical radiological pattern**	10 (29.4)	26 (81.3)	0.14	14 (38.9)	56 (82.4)	0.62
***Pj*** ** biological criteria**						
**Positive microscopic examination (n (%))**	0 (0.0)	4 (12.5)	0.11	0 (0.0)	3 (4.4)	0.55
**Median fungal load (Cq) [IQR]**	33.4 [32.0; 35.3]	30.8 [25.2; 34.7]	0.01	35.4 [33.2; 36.9]	31.2 [28.1; 33.9]	<0.0001
**Median fungal load (genome copies/mL) [IQR]**	5481 [1267; 13,898]	32,179 [4695; 2,499,000]	0.0079	1256 [644; 4677]	26,714 [4826; 322,155]	<0.0001
**Anti-*Pj* treatment** **after sampling (n (%))**						
**Curative**	9 (26.5)	26 (81.3)	<0.0001	10 (27.8)	55 (80.9)	<0.0001
**Prophylactic**	6 (17.6)	2 (6.3)	0.26	5 (13.9)	6 (8.8)	0.42
**Outcome** **(number of deaths, (%))**	6 (17.6)	13 (40.6)	0.10	4 (11.1)	12 (17.6)	0.57

SOT: solid organ transplant recipients; IQR: interquartile range; CTC: corticosteroids; Cq: quantification cycle; *: including 1 patient with allogeneic hematopoietic stem cell transplantation (HSCT); **: including 1 patient with allogeneic HSCT.

**Table 2:** qPCR threshold values with corresponding performances.

Sample Type	Cq	Fungal Load (Genome Copies/mL)	Sensitivity (%)	Specificity (%)
**BAL**	25.43	1,860,000	25.0	**100**
	**31.45**	**8275**	**59.4**	**82.4**
	37.08	396	**100**	2.9
**URT**	22.55	4,120,000	7.4	**100**
	**32.33**	**8130**	**63.2**	**88.9**
	38.21	264	**100**	2.6

Cq: quantification cycle.

## Data Availability

Data supporting the results of this study shall, upon appropriate request, be available from the corresponding author.

## Appendix A

**Table A1:** Characteristics of PCP patients according to respiratory sample types and PCP subgroups.

	BAL (n = 32)				URT (n = 68)			
	Proven PCP (n = 4)	Probable PCP (n = 17)	Putative PCP (n = 11)	*p*	Proven PCP (n = 4)	Probable PCP (n = 46)	Putative PCP (n = 18)	*p*
**Median age (years)** **[min–max]**	63 [54–83]	69 [37–83]	68 [53–80]	ns	66 [59–83]	69 [30–90]	71 [59–94]	ns
**Sex ratio** **M/W (n/n)**	0.33 (1/3)	1.13 (9/8)	0.8 (5/6)	ns	1 (2/2)	1.19 (25/21)	1.25 (10/8)	ns
**Immunosuppressive condition (n (%))**								
**SOT**	1 (25)	4 (23.5)	1 (9.1)	NA	0 (0)	17 (37.0)	6 (33.3)	ns
**Hematologic malignancy**	0 (0)	4 (23.5)	2 (18.2)	ns	1 (25)	6 (13.0)	2 (11.1)	NA
**Solid malignancy**	3 (75)	5 (29.4)	7 (63.6)	ns	3 (75)	9 (19.6)	6 (33.3)	*
**Inflammatory disease**	0 (0)	3 (17.7)	1 (9.1)	ns	0 (0)	7 (15.2)	4 (22.2)	ns
**Other**	0 (0)	1 (5.9)	0 (0)	NA	0 (0)	7 (15.2)	0 (0)	NA
**Total lymphocytes count** **G/L [IQR]**	0.37 [0.22–0.64]	0.46 [0.21–0.69]	0.76 [0.54–1.01]	ns	0.50 [0.22–1.98]	0.84 [0.42–1.16]	0.66 [0.37–1.03]	ns
**CD4 T lymphocytes count < 200 cells/mm^3^** **(n/total (%))**	0/1 (0)	5/7 (71.4)	2/4 (50)	ns	1/2 (50)	9/17 (52.9)	3/6 (50)	ns
**Use of CTC** **(n (%))**	3 (75)	15 (88.2)	5 (45.5)	*	2 (50)	33 (71.7)	10 (55.6)	ns
**Clinical/radiological features** **(n, (%))**								
**Hypoxemia**	4 (100)	7 (41.2)	8 (72.7)	ns	3 (75)	28 (60.9)	8 (44.4)	ns
**Dyspnea**	4 (100)	10 (58.8)	9 (81.8)	ns	4 (100)	37 (80.4)	16 (88.9)	ns
**Fever (>38.3 °C)**	4 (100)	9 (52.9)	8 (72.7)	ns	3 (75)	36 (78.3)	7 (38.9)	**
**Dry cough**	0 (0)	5 (29.4)	3 (27.3)	NA	0 (0)	13 (28.3)	7 (38.9)	ns
**Typical radiological pattern**	4 (100)	17 (100)	4 (36.3)	NA	4 (100)	46 (100)	5 (27.8)	NA
***Pj*** ** biological criteria**								
**Positive microscopic examination (n (%))**	4 (100)	0 (0)	0 (0)	NA	4 (100)	0 (0)	0 (0)	NA
**Median fungal load (Cq) [IQR]**	21.2 [19.4; 29.1]	32.0 [28.9; 34.3]	30.2 [29.0; 35.6]	ns	25.7 [20.4; 33.1]	31.1 [28.1; 34.0]	32.1 [28.2; 33.9]	ns
**Median fungal load (genome copies/mL) [IQR]**	15,940,000 [1,729,000; 39,160,000]	24,161 [5986; 117,301]	37,968 [776; 81,525]	ns	8,292,000 [8540; 22,890,000]	32,496 [6435; 220,878]	13,078 [2144; 287,050]	ns
**Anti-*Pj* treatment after sampling** **(n (%))**								
**Curative**	4 (100)	14 (82.4)	8 (72.7)	NA	4 (100)	40 (87.0)	11 (61.1)	*
**Prophylactic**	0 (0)	2 (11.8)	0 (0)	NA	0 (0)	5 (10.9)	1 (5.6)	NA
**Outcome (number of deaths, (%))**	1 (25)	7 (41.2)	5 (45.5)	ns	1 (25)	9 (19.6)	2 (11.1)	NA

SOT: solid organ transplant recipients; IQR: interquartile range; CTC: corticosteroids; Cq: quantification cycle; ns: not significant; *: *p* < 0.05; **: *p* < 0.01; NA: not applicable (low number of patients in different categories).
